# Methods Used in the Spatial and Spatiotemporal Analysis of COVID-19 Epidemiology: A Systematic Review

**DOI:** 10.3390/ijerph19148267

**Published:** 2022-07-06

**Authors:** Nushrat Nazia, Zahid Ahmad Butt, Melanie Lyn Bedard, Wang-Choi Tang, Hibah Sehar, Jane Law

**Affiliations:** 1 School of Public Health Sciences, University of Waterloo, 200 University Ave West, Waterloo, ON N2L 3G1, Canada; zahid.butt@uwaterloo.ca (Z.A.B.); melanie.lyn.bedard@uwaterloo.ca (M.L.B.); wc4tang@uwaterloo.ca (W.-C.T.); hsehar@uwaterloo.ca (H.S.); jane.law@uwaterloo.ca (J.L.); 2 School of Planning, University of Waterloo, 200 University Ave West, Waterloo, ON N2L 3G1, Canada

**Keywords:** clustering analysis, spatial association, systematic review, COVID-19, Bayesian methods

## Abstract

The spread of the COVID-19 pandemic was spatially heterogeneous around the world; the transmission of the disease is driven by complex spatial and temporal variations in socioenvironmental factors. Spatial tools are useful in supporting COVID-19 control programs. A substantive review of the merits of the methodological approaches used to understand the spatial epidemiology of the disease is hardly undertaken. In this study, we reviewed the methodological approaches used to identify the spatial and spatiotemporal variations of COVID-19 and the socioeconomic, demographic and climatic drivers of such variations. We conducted a systematic literature search of spatial studies of COVID-19 published in English from Embase, Scopus, Medline, and Web of Science databases from 1 January 2019 to 7 September 2021. Methodological quality assessments were also performed using the Joanna Briggs Institute (JBI) risk of bias tool. A total of 154 studies met the inclusion criteria that used frequentist (85%) and Bayesian (15%) modelling approaches to identify spatial clusters and the associated risk factors. Bayesian models in the studies incorporated various spatial, temporal and spatiotemporal effects into the modelling schemes. This review highlighted the need for more local-level advanced Bayesian spatiotemporal modelling through the multi-level framework for COVID-19 prevention and control strategies.

## 1. Introduction

Coronavirus disease 2019 (COVID-19) is a highly transmittable and pathogenic viral infection caused by severe acute respiratory syndrome coronavirus 2 (SARS-CoV-2) [1]. COVID-19 has caused a global pandemic and has contributed to many deaths worldwide, posing a massive threat to global public health and the economy that may take several years to recover [2]. As of 13 March 2022, over 452 million confirmed and over 6 million deaths have been reported worldwide from COVID-19 [3]. COVID-19 incidence and risk can vary in space and time, and it is important to understand these variations [4,5]. With the increasing availability of high-quality data and improved computational capabilities, numerous geospatial methods and tools have been developed and used in infectious diseases, including COVID-19 surveillance [6,7]. These spatial tools have been used to investigate an outbreak using both points and aggregated datasets [8]. A variety of retrospective studies reporting spatial dynamics of COVID-19 have been published that highlight the spatial and spatiotemporal fluctuations of COVID-19.

In spatial epidemiology, spatial clustering analysis plays an important role in identifying spatial aggregation of disease cases by identifying whether geographically grouped cases can be explained by chance or are statistically significant to find evidence of etiologic factors [9,10]. Past studies have demonstrated that the distribution of infectious diseases is often determined by different social processes related to the space of occurrence [8,11]. The complex interaction of different determinants such as socioeconomic vulnerability, rapid population growth, and urbanization combined with environmental variables can result in spatial and spatiotemporal variations in COVID-19 infections [12]. Spatial analysis and identification of areas with COVID-19 clusters, followed by the characterization of the drivers of the dynamics in these clusters, has been promoted to carry out an investigation of outbreaks [13,14]. The resulting maps from these spatial methods can help prevent and control cases with targeted public health action plans and guided interventions in areas with higher than expected disease risk while motivating the population with various public health programs with the advanced knowledge of disease etiological characteristics [14].

In spatial modelling, frequentist and Bayesian methods are the two schools of thought of statistical inference, employed primarily to identify high-risk clusters or hotspots using clustering analysis and to identify risk factors using spatial regression modelling techniques [15]. The traditional frequentist approach is based on the likelihood function to derive the parameter estimates [16]. On the contrary, the Bayesian approach uses probability to measure the uncertainties in estimates, prediction or inference on posterior distributions by incorporating spatial and temporal dependencies by specifying a prior [17,18]. These priors can be used to incorporate prior knowledge from preceding studies [19]. Bayesian spatiotemporal models have been beneficial and provided more variations to incorporate spatial and spatiotemporal structures to describe epidemiological data effectively [20,21].

While spatial analysis is limited to spatial variations, spatiotemporal analysis can investigate the space-time variation by identifying disease patterns persisting over time over spatial units, characterized by incorporating spatial and spatiotemporal structures. In addition to the model types, the spatial unit of the data is also an important determinant of the cluster patterns and the relevant associations [20]. Regardless of the true extent of spatial correlation, different spatial resolutions can lead to different results for the same dataset [20,21]. The effects observed at global or regional levels may not hold at the local or individual levels, causing ecological fallacy [21,22].

To the best of our knowledge, hardly any study has conducted a detailed systematic review of the spatial and spatiotemporal methods in COVID-19 research. A review by Franch-Pardo et al., (2020) has summarized the geospatial methods used during the early stages (January–May 2020) of the pandemic [23]. A review by Fatima et al., in 2021, using data until September 2020, conducted a scoping review of the methods and associated findings in relation to COVID-19 and sociodemographic and environmental characteristics [24]. These past reviews lack a comprehensive review of the spatial and spatiotemporal methodological frameworks, models and covariates used in modelling COVID-19 infection. In addition, despite the popularity of Bayesian models in spatial epidemiology, the Bayesian models were not thoroughly discussed in these past reviews. 

The objective of this study was to systematically review the spatial and spatiotemporal methods used to identify spatial variations of COVID-19 incidences and associated socioeconomic, demographic and climatic risk factors for such spatial variations. Our study aims to gain more value from such spatial analytical tools to help improve research designs by identifying the gaps in research that can be used to make recommendations for improving practice and identifying opportunities for further development in this area. 

The rest of the paper is structured as follows. In the next section, we describe the methodologies followed to conduct the systematic review in accordance with the Preferred Reporting Items for Systematic Reviews and Meta-Analyses (PRISMA) guidelines. The following section presents the results from the systematic review. Finally, findings from this systematic review are assessed in the discussions section. The spatial methodologies used to answer the research questions of this review and the gaps in the existing studies, and the direction of future research are discussed in this section. 

## 2. Materials and Methods

### 2.1. Data Source and Search Strategy

The methodology of this review was conducted in accordance with the Preferred Reporting Items for Systematic Reviews and Meta-Analyses (PRISMA) guidelines [25]. Our search strategy aimed to identify peer-reviewed studies of the distribution and determinants of COVID-19 that employed spatial clustering and spatial regression methods. In this review, studies were considered spatial if they incorporated any spatial or spatiotemporal approaches such as cluster detection methods, spatial risk modelling and spatial regression analysis with socioeconomic, demographic, or climatic variables. We employed a broad search strategy of four electronic databases: Embase, Scopus, Medline, and Web of Science. The search was run from 1 January 2019 to 7 September 2021, using a combination of keywords based on our concepts of spatial clustering and spatial regression analysis on COVID-19. We acquired and used search strings for COVID-19 developed and peer-reviewed by Research Information Specialists at CADTH [26]. The full search strategy is provided in the Appendix A. 

### 2.2. Eligibility, Inclusion and Exclusion Criteria

We have used Covidence [27], a web-based software platform for systematic review, to perform our screening and data extraction process. Each included study was reviewed, and information about the study was extracted. After excluding duplicates, titles and abstracts of each study were screened independently by at least two independent reviewers (NN and HS, or WT and MB) to identify potentially eligible studies. We included studies that incorporated a geospatial method to observe the spatial risk of COVID-19 and studies that performed a spatial regression analysis to understand the association between COVID-19 and socioeconomic, demographic, or climatic covariates. We only included studies in English, and no exclusions were made on the basis of location. The articles were limited to COVID-19 in humans. Studies targeting specific population groups such as the elderly or children, as well as the studies that considered the entire population, were accepted. Studies were excluded if they utilized non-spatial or purely mathematical models, reported only the temporal patterns of COVID-19, the covariate in the regression models were not related to research questions and contained insufficient information on spatial methods. Articles were also excluded if not peer-reviewed, the study design was not an analytical observational or cross-sectional study design, and the article was a correspondence letter, conference paper, opinion piece or a review. After the title and abstract screening, the identified papers were independently evaluated by thoroughly reading the full text by two independent reviewers and selected according to the same inclusion criteria. The conflicts in the screening process were resolved by a third reviewer (ZAB).

### 2.3. Data Extraction and Synthesis

All data from each article were extracted and collected manually by at least two independent reviewers and stored in a Microsoft Excel 365 spreadsheet. Extracted data included first author name, year of publication, study area, study units, length of study, COVID-19 data description, covariate data, spatial methodology, type of analysis (Bayesian or Frequentist) and visualization techniques. Furthermore, methodological details such as spatial models, model selection criteria, model structure (spatial, temporal and space-time effects), relative risk estimation, model inference approaches, or sensitivity analysis for the priors were also collected. 

### 2.4. Quality Assessment

All of the included studies were scored to assess the study bias using the Joanna Briggs Institute (JBI) risk of bias tool for prevalence studies [28]. The checklist comprises nine questions with binary scores of 0 or 1 for each question (yes or no). There is no specific score specified for excluding studies by The JBI tool. We believe it is appropriate to include all of the studies while accounting for the potential risk of bias in those with lower scores. The assessment of the quality of all included studies was conducted independently by two reviewers to quantify the scoring and overall evaluation of quality. Any disagreement was resolved through discussion between the first and second reviewers for each article.

## 3. Results

### 3.1. Literature Search

**Figure 1:**
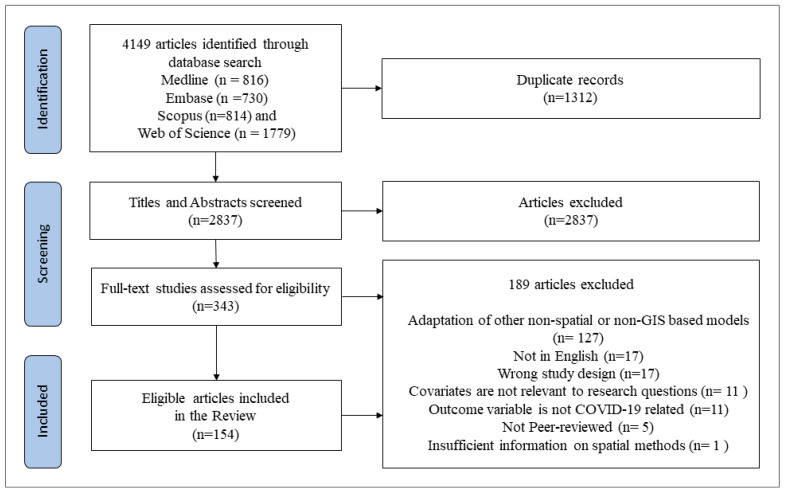
Study inclusion flow Chart (PRISMA).

Figure 1 depicts the PRISMA flow chart of the literature selection process, and the detailed contents of the selected articles are provided in Supplementary File S1. We obtained 816 articles from Medline, 730 articles from Embase, 814 articles from Scopus and 1779 articles from Web of Science. Out of the 4149 articles initially identified, 1312 duplicate studies were removed, leaving 2837 articles for screening. An additional 2487 articles were removed during the title and abstract screening process. Furthermore, 186 of the 354 remaining articles were excluded for not meeting the inclusion criteria during the full-text screening process. Finally, a total of 154 articles were included in this review.

### 3.2. General Characteristics of the Selected Studies

All of the studies used secondary observational data on COVID-19 cases collected from the national registries. Case incidence (*n* = 141) was more commonly studied than mortality (*n* = 16). Three studies included both incidence and mortality from COVID-19. Nearly all of the studies (*n* = 150) used COVID-19 data of all ages except four studies that used COVID-19 data for specific population groups: children (*n* = 1), 60 years and older (*n* = 1), 18 years and older (*n* = 1), and indigenous population (*n* = 1) to perform the analysis (Supplementary File S1).

### 3.3. Time Intervals and Geographic Regions

**Figure 2:**
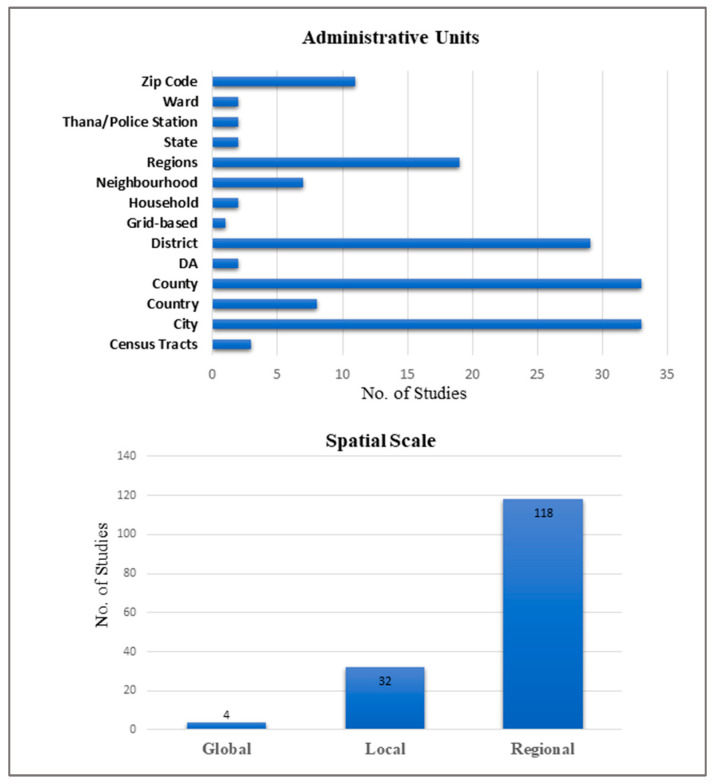
Geographical units, spatial scale and length of studies (*n* = 154).

**Figure 3:**
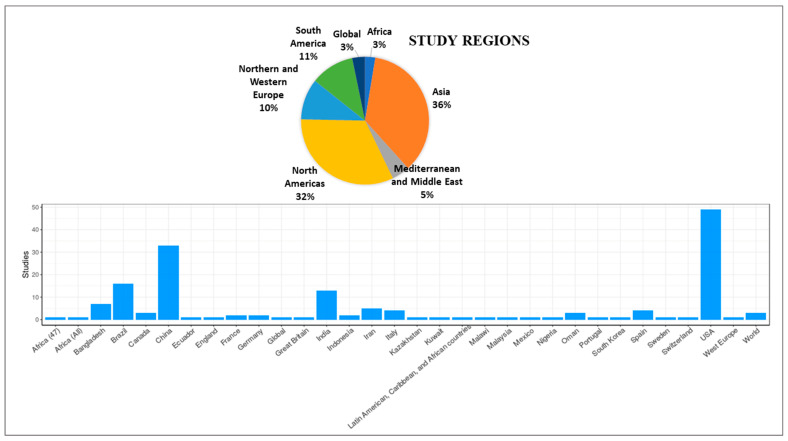
Locations of the study area using spatial methods by region and country (*n* = 154).

Reviewed articles used COVID-19 data ranging from 1 week to 15 months, with a median of 4 months. A total of 24 (15%) studies used COVID-19 datasets with <1 month, 124 (80%) studies used datasets <6 months, while 6 (4%) studies used datasets of >12 months. Moreover, 118 (76%) studies used the regional level spatial scale, 32 (21%) studies used the local spatial scale, and 4 (2.5%) studies used the global spatial scale in performing the analysis (Figure 2). The majority of the study area was conducted in Asia (36%), followed by North America (32%). More than half of the studies originated from China (*n* = 33) and the USA (*n* = 49) (Figure 3).

### 3.4. Data Used and Scale of Analysis

The spatial analysis was mostly performed using aggregated data at an administrative unit. However, the scale of aggregation varied widely. Zip codes were used in 11 studies, regions in 19 studies, neighborhoods in 7 studies, districts in 29 studies, cities in 33 studies and counties in 33 studies. Two studies analyzed data at the household level, and one study performed a grid-based analysis, while eight studies aggregated the data at the county level (Figure 2). Only one study aggregated the data in space-time to generate a special data structure where x and y dimensions represent space and t dimension represents time using ArcGIS software [29].

### 3.5. Study Design Perspective

Ecological studies are a form of study design where the unit of analysis is not grouped by an individual but rather grouped by a unit of analysis such as the county, zip code or city [30]. Ecological studies often incorporate spatial designs and analysis. Most of the studies (*n* = 152) in this review were ecological studies where the data were aggregated at a spatial unit. Only two studies were conducted at the household level. 

### 3.6. Software

The GIS software used in those studies are ArcGIS Desktop/ArcGIS Pro (*n* = 43), GeoDa (*n* = 27), SaTScan (*n* = 29), QGIS (*n* = 4), and R (*n* = 20). Several other software packages such as GWR4, BayesX (*n* = 1), and WinBUGS (*n* = 1) were also used for specific spatial analysis. 

### 3.7. Methods Used to Identify Spatial Variations of COVID-19 and Associated Risk Factors

Out of the 154 selected studies, 132 studies reported spatial variations of COVID-19 incidence and 67 studies identified associated risk factors for such variations. Most of the studies used frequentist methods, while 24 (15%) studies used Bayesian methods. A total of 49 (32.8%) studies observed spatial heterogeneity in the disease risk using global Moran *I*. The most frequently used method was local Moran’s *I* (*n* = 46), followed by Getis-Ord Gi* statistic (*n* = 36), Kulldorff’s spatial scan statistic (*n* = 34) and Kernel density (*n* = 9). Of the 34 studies using Kulldorff’s scan statistic, 10 studies analyzed the data to identify spatial clusters, while 24 analyzed the data to identify spatiotemporal clusters. One study used Kulldorff’s multivariable permutation scan statistic (MPSS) by accounting for socioeconomic variables [31]. Other frequentist clustering methods such as k-means cluster (*n* = 2), Ripley’s K function (*n* = 1), MST-DBSCAN (*n* = 1), and spatiotemporal event sequence-based clustering (*n* = 1) were also reported.

The most frequently used method used to identify the drivers of spatial variations of COVID-19 was the geographically weighted regression (GWR) (*n* = 36), which was used to model the local association between predictors and COVID-19, followed by the spatial regression models (*n* = 20), such as spatial error model (SEM) and spatial lag model (SLM) to identify the global association by introducing spatial context. Four studies used a recently developed Geodetector Q statistic method that detected spatial heterogeneity of COVID-19 cases and identified the potential drivers for these variations (Table 1). Many of these studies used more than one type of analysis. The summary of the methods used in each of these studies is presented in Supplementary File S1. 

### 3.8. Spatial Interpolation Methods

Spatial interpolation is the process of mapping a variable by interpolating point or area data with known values to estimate values at unknown points or areas, based on the assumption that objects that are closer in proximity are spatially correlated [182]. The spatial interpolation methods also allow cross-validation statistics to determine how the interpolation models fit the data. A total of 5 studies used spatial interpolation, such as Inverse distance weighting (IDW) (*n* = 2), Thiessen polygon method (*n* = 1), areal interpolation (*n* = 1), and local empirical Bayesian method (*n* = 6), to create smoothed surfaces of the spatial risk of COVID-19. Oluyomi et al. [151] implemented the area interpolation tool in ArcGIS Pro 2.6 using a k-Bessel model to visualize and obtain predicted values of COVID-19 incidence at the census tract level. While Nasiri et al. [107] used the IDW method to create interpolated maps of infected COVID-19 patients across Tehran, Iran. Ramírez and Li [164] used the IDW algorithm to interpolate and create a 3D continuous surface of hotspots of COVID-19 incidence across counties in the USA at five-time points. Arif et al. [165] used the Theissen polygon method, the nearest neighbor interpolation method, to create interpolated polygon maps that showed the spatial distribution of COVID-19 cases in the southern states of India and examined the association between COVID-19 cases and population density. Six studies used the local empirical Bayesian smoothing technique to get spatially smoothed rates of COVID-19 incidence in each spatial unit of analysis [38,62,78,83,94,113]. The smoothing technique helped reduce the extreme variations in incidence rates between neighboring areas. In contrast to the standard Bayesian methods, where prior distribution is fixed before analysis, the empirical Bayesian method estimates the prior distribution from the data [183].

### 3.9. Spatial Statistical Models (Frequentist)

Sun et al. [64] examined associations of COVID-19 mortality rate with socioeconomic and environmental factors across England using a spatial autoregressive (SAR) model and chose the matrix exponential spatial specification (MESS) and fast random effects eigenvector, spatial filtering models. The MESS specification uses an exponential decay pattern in the influence of the high-ordering neighboring relationship for the spatial autoregressive process. 

Kindi et al. [118] observed the association between different demographic and socioeconomic factors of COVID-19 in Oman using a Generalized Linear Model (GLM) to understand and predict early incidence and infection rates, using an individual regression equation to describe the process. Oluyomi et al. [151] adopted a Poisson-based regression model using a Poisson-gamma mixture distribution that allows for extra variations to understand the association between social determinants of health and community-level COVID-19 case counts. The model also used population as an offset term referred to as the exposure variable. The relative risk of COVID-19 was estimated by exponentiating the regression coefficient and mapped to show the variations in the risk. Chien et al. [162] used a Poisson-based distributed lagged nonlinear model with a spatial function to evaluate the impact of weather variability using meteorological factors such as temperature, relative humidity and precipitation on COVID-19. The model used a maximum lag of 14 days to consider the COVID-19 incubation period and the spatial correlation was controlled by adding a two-dimensional spatial function that accounts for the spatial coordinate in latitude and longitude. The relative risks were estimated and mapped by transforming the coefficients from the outputs. 

Feng et al. [163] used a spatial-temporal generalized additive model (GAM) to model the COVID-19 mortality risk in Toronto, Canada. Non-linear and spatial-temporal interaction effects of population density and average income were modelled as a two-dimensional spline smoother to reflect how the spatial pattern of mortality risk evolved over time. Gaudart et al. [43] used a GAM model negative binomial regression and gaussian kriging smoothing technique to identify the factors associated with the spatial heterogeneity of COVID-19 in France during the first wave. The gaussian kriging accounts for the spatial autocorrelation using aspatial smoother based on geographic coordinates for the administrative units. The log of the population was used as an offset variable.

### 3.10. Bayesian Spatial and Spatiotemporal Statistical Models

A total of eighteen studies used the Bayesian generalized linear mixed models (GLMM) over space and time. The variations of risk in space and time were modelled in 16 studies using the Poisson-based models, and the random effects were used to account for the extra Poisson variability. Table 2 shows a summary structure of the spatial and spatiotemporal structure of the Bayesian models.

***GLMM with spatial random effects:*** A GLMM model with only spatial random effects has been used in five studies [166,167,168,169,170]. The general model is expressed as:

(1) $$Y_{i}\sim \;Poisson\left(E_{i}\theta_{i}\right)$$

(2) $$\log(\theta_{i})=\alpha +\beta x+u_{i}+v_{i}$$

where $Y_{i}$ is the observed case or death counts in an area *i,* $E_{i}$ presents the expected case count, $\theta_{i}$ the relative risk, α is the intercept, β represents the coefficient of the covariates, $v_{i}$ is the spatially unstructured random effect term that captured normally distributed or Gaussian random variation around the mean or intercept, and $u_{i}$ is the spatially structured conditional autoregressive term.

The Besag-York-Mollié (BYM) model [184] is a lognormal Poisson model originally developed for disease mapping and was most commonly adopted (11 out of 18 Bayesian studies) for the overall spatial component in our studies [166,167,169,170,171,172,173,174,176,177,180]. A BYM model is a lognormal Poisson model developed for disease mapping that includes both an ordinary random-effects component to account for non-spatial heterogeneity and an ICAR component for spatial smoothing [185]. The BYM model [185] is modelled in Equation (3) as:

(3) $$n_{i}=\mu +x\beta +\emptyset +\theta$$

where *n_i_* is the log relative risk for zone *i*, *μ* is the fixed intercept, *x* is the matrix of explanatory spatial covariates, *β* is vector of regression coefficients which are constant across all regions, ∅ is an ICAR spatial component and *θ* is an ordinary random effects component for non-spatial heterogeneity.

Whittle et al. [168] additionally used the BYM2 model proposed by Riebler et al. [186] that reparametrizes the BYM model and uses a scaled spatial structured and unstructured component, making parameters interpretable. While four of these GLMM models used Poisson-based modelling, Millett et al. [169] used a zero-inflated negative binomial model with a logarithmic link function. A number of covariates have been incorporated in modelling these GLMM models with spatial effects. DiMaggio et al. [167] included zip code level explanatory variables for the proportion of persons identifying as black/African American, with COPD, heart disease, older than 65 years, a measure of housing density in the model. Whittle et al. [168], Millett et al. [169], and Yang et al. [170] have used various socioeconomic predictors, demographic or housing covariates to model the spatial risk of COVID-19.

***GLMM with spatial and temporal random effects:*** Among the temporal components in our models, the Gaussian random walk model of order 1 (RW1) or order 2 (RW2) was more commonly used in eight studies [112,171,172,173,174,175,176,177]. The RW1 [187,188] model on the set of time-point-specific is expressed in Equation (4). For *t* = 2,…..., *T*,

(4) $$v_{t}=v_{t-1}+\varepsilon_{t}$$

where $\varepsilon_{t}\;\sim \;N\left(0,\sigma^{2}\right)$ represents the noise term, and $\varepsilon_{2},\ldots ,\varepsilon_{T}$ are independent. 

In the RW2 model, an extended to a higher-order version of the RW1 model generally yields a smoother temporal pattern by assigning more neighbors to each time point. Equation (2) specifies the RW2 model on a set of temporal parameters with *T* ≥ 3 in Equation (5) [189]. For *t* = 3,…., *T*

(5) $$v_{t}=2v_{t-1}-v_{t-2}+\varepsilon_{t}$$

Blangiardo et al., 2020 [172] and Jalilian et al., 2021 [174] have both used Poisson-based GLMM models with spatial, temporal random effects and temporal covariates. While these two studies have used the BYM model for the overall spatial random effects, the temporal random effects have followed the random walk models.

The basic formula used by Blangiardo et al., 2020 [172] is given in Equation (6) as:

(6) $$\log(\rho_{ijtk})=\beta_{0k}+u_{i}+v_{i}+\omega_{jt}+f(x_{it})$$

where $\rho_{ijtk}$ is the mortality relative risk created by summing across the age groups for each municipality and year and then dividing by the total number of weeks in each year. The year-specific intercept is defined as $\beta_{0k}=\beta_{0}+\varepsilon_{k}$, where $\beta_{0}$ is the global intercept, ($\left(u_{i}+v_{i}\right)$ is the BYM specification), $\omega_{jt}$ is the weekly random effect through RW1. 

The log-linear model by Jalilian et al., 2021 [174] has incorporated population density in the model with the BYM model for spatial components and RW2 models for the temporal trends. The formula is expressed as:

(7) $$\theta_{it}=\exp(\mu +\beta d_{i}+\delta_{t}+\varepsilon_{t}+\xi_{t})$$

where, μ in the intercept, $d_{i}$ population density of region *I*, β is the regression coefficient,$\;\delta_{t}$ represents the temporal trend, $\varepsilon_{t}$ accounts for temporal correlation., $\xi_{t,}\;\zeta_{t}$ explains spatial correlation spatial effects. 

***GLMM with spatial, temporal random effect and spatiotemporal random effects:*** A total of eight studies have used a GLMM model with spatial, temporal and spatiotemporal random effects in the modelling scheme. These models incorporated a space-time interaction term $\delta_{it}$ Introduced by Knorr-Held (2000) [189]. The general formula is given below:

(8) $$\log\left(\theta_{it}\right)=\alpha +u_{i}+v_{i}+\gamma_{t}+\emptyset_{t}+\delta_{it}$$

where $Y_{i}$ is the observed case or death counts in an area *i,* $E_{i}$ presents the expected case count, $\theta_{i}$ the relative risk, α denotes the intercept of the model ($u_{i}$ and $v_{i}$) the BYM components for the overall spatial structure and $\gamma_{t}$ and $\emptyset_{t}$ are the structured and unstructured temporal random effects, respectively, $\delta_{it}$ is the random spatiotemporal effect. 

While the majority of the studies used the BYM modelling for the spatial structures, Ngwira et al., 2021 [177] used Leroux CAR (LCAR), proposed by Leroux et al., is a variation of the BYM and CAR model, as the conditional distribution is specified in such a way that it incorporates characteristics of both structured and unstructured random effects (from BYM model) into a single parameter [190].

Spatiotemporal studies by Wang et al. [160], Briz-Redón et al. [173] and Jaya et al., 2021 [175] did not incorporate any covariates to measure the spatiotemporal relative risk of COVID-19. Bermudi et al. [171], Ngwira et al., 2021 [177] and Paul et al., 2021 [178] incorporated socioeconomic covariates, while Johnson et al., 2021 [176] included 6 Social vulnerability and 7 environmental variables as fixed effects.

Paul et al., in 2020 [112] have fitted a Bayesian spatiotemporal model to county-level demographics, smoking rates, and chronic diseases, incorporating a latent autoregressive Gaussian space-time process with covariance matrix characterized by exponential covariance function using geodesic distances between county centroids.

While most of these spatiotemporal models followed Poisson-based hierarchical modelling, a study by Paul et al., in 2021 [178] adopted a Bayesian semi-parametric spatiotemporal negative binomial modelling.

***Geo-additive hurdle Poisson Model***

Gayawan et al. [180] used a two-component geo-additive hurdle Poisson model structured spatial and spatiotemporal effects to simultaneously analyze the zero counts and the frequency of occurrence of COVID-19 cases. The expected value of *Y* is given by E(Y)=pμ/(1−exp(−μ)). For an identically distributed random variable, the hurdle Poisson distribution is expressed as:

$$P\left(Y_{i}=y|p,\mu \right)=\{\begin{array}{c} p\\ \left(1-p\right) \end{array}\begin{array}{cc} & y=0\\ \frac{\mu^{y}(\exp\left(-\mu \right)}{y!\left(1-\exp\left(-\mu \right)\right)} & y=1,2,\ldots ,\infty \end{array}$$

$Y_{i}$ is the response variable of interest, *p* is the none occurrence probability, μ is the frequency of occurrence. 

The geo-additive hurdle Poisson model is given by

$$\{\begin{array}{c} g_{1}^{-1}\left(p\right)=\eta_{i}^{P}=\beta_{0}^{P}+S_{str}^{P}+S_{unstr}^{P}+T^{P}+{\left(ST\right)}^{P}\;\\ g_{2}^{-1}\left(\mu \right)=\eta_{i}^{\mu }=\beta_{0}^{\mu }+S_{str}^{\mu }+S_{unstr}^{\mu }+T^{\mu }+{\left(ST\right)}^{\mu } \end{array}$$

where $g_{1}$ and $g_{2}$ are link functions chosen as logit and log links for the parameters, and $S_{str}^{P},\;S_{unstr}^{P}$ are the structured and unstructured random effects, $T^{P}$ is the temporal random effect, and ST is the space-time interaction effect. 

***GLM with Bayesian model averaging (BMA):*** Olmo et al. [181] applied the Bayesian model averaging (BMA) technique using a Poisson generalized linear model (GLM) that included a set of demographic and socioeconomic covariates. The BMA technique [191] estimates all of the candidate models and computes a weighted average of the estimates while taking the uncertainties of the models into account. The spatial effects were incorporated using the autoregressive (SAR) model [192]. The temporal effects were captured by an autoregressive lag of the response variable ad the lagged incidence rate.

***GLMM with Separable Gaussian spatiotemporal process:*** Rawat et al., 2021 [179] proposed a model structure that includes a separable Gaussian spatial-temporal process model implemented through a Bayesian framework, in conjunction with an additive mean structure and a random error process to estimate the relative risk of COVID-19. The spatial and temporal trends both follow an exponentially decaying pattern. This proposed approach provided short-term and long-term predictions for the COVID-19 response variable for any spatial location, even if it was unobserved in the data. 

### 3.11. Relative Risk Estimation

Sixteen studies quantified the relative risk of COVID-19 to identify whether an area had a higher or lower risk than the average risk across space and time based on the posterior predictive distributions of the Bayesian models. The determinants of the relative risks were implicitly captured by the random effects in the models. Geographic areas with relative risk greater than one were generally identified as hotspots or high-risk areas. 

### 3.12. Bayesian Model Selection

The Deviance Information Criteria (DIC) values introduced by Spiegelhalter et al. [193] were most commonly used (11 studies) to measure the goodness of fit of various Bayesian models in to compare the performances of various Bayesian models in a study [166,167,168,170,171,173,174,175,176,177,180]. Five studies have also used the Watanabe information criterion (WAIC), proposed by Watanabe and Opper [194], to select the best model [173,174,175,178]. The models with the lowest DIC or WAIC values were chosen as the best-adjusted models. Some of the other model selection criteria used in the studies were the Bayesian cross-validation criterion (BCV) [174], mean absolute percentage error (MAPE) [179], Root Mean Squared Error (RMSE) [179], Continuous Ranked Probability Score (CRPS) [179], highest probability (HPM) [181], and best prediction (BPM) [181] to select the best model (Table 2). Results reported were generally based on the best model selected using these criteria.

### 3.13. Model Implementation

The recently developed R software package INLA (Integrated Nested Laplace Approximation) was the commonly used (14 out of the 18 Bayesian studies) approach to perform the Bayesian models. INLA is an alternative method to the traditional MCMC. Compared to the traditionally fitted model through the exact method of the Markov Chain Monte Carlo (MCMC) sampling method, INLA uses a combination of analytical approximations and numerical algorithms to approximate the posterior distributions [195]. A total of four studies adopted the MCMC method to fit the Bayesian hierarchical models. 

### 3.14. Sensitivity Tests of Priors

Out of the 18 Bayesian statistical studies, only 4 studies used a sensitivity analysis of the priors. A sensitivity analysis of priors is essential to understand the impact of the prior on the latent classes, whether diffuse or informed priors are implemented [196]. Gayawan et al. [180], Johnson et al. [176], Ngwira et al. [177], and Yin et al. [197] examined different prior and hyperprior specifications and performed sensitivity analyses and found that their results were not sensitive to their choice of priors. 

### 3.15. Factors Associated with the Risk for COVID-19

In this systematic review, three categories of covariates were identified from the spatial regression models to have a significant influence on COVID-19, namely climatic, demographic, and socioeconomic covariates. In the frequentist models, significant covariates were generally determined if their *p*-values were less than 0.001, 0.05 and 0.01 in the frequentist regression models. In the Bayesian regression framework, the variable was considered influential if the 95% CI of the corresponding relative risk (exponentiated of beta coefficients) did not include one. The observed spatial patterns of COVID-19 were consistently positively linked to population density (*n* = 22) and the aging population (*n* = 15). However, six studies have found a negative association between COVID-19 and the aging population. Ethnicity or minority statuses such as percentage of Black (*n* = 12), Hispanic (*n* = 3), Native American (*n* = 3), Asian (*n* = 3), and immigrants (*n* = 2) were also found to be positively associated with the risk for COVID-19. Commonly associated socioeconomic covariates included education (*n* = 4), income/poverty level (*n* = 13), and social vulnerability/deprivation/income inequality index (*n* = 6). Temperature (*n* = 5), relative humidity (*n* = 3), land surface temperature (*n* = 2) and wind speed (*n* = 3) were some of the common climatic covariates that were found to have an association with COVID-19. (Table 3) The details of the regression variables for each study are provided in the Supplementary File S2.

### 3.16. Assessment of Quality

Based on the JBI risk of bias, the assessment scores of our studies ranged from 7 to 9 out of 9. A few of the studies in this review lacked details of the study area, descriptions of the datasets or methods. The median score across 154 studies was high, 9 out of 9. The detailed quality assessment scores of these 154 studies are included in Supplementary File S3. 

## 4. Discussion

In this study, we reviewed 154 published peer-reviewed articles on COVID-19 that applied various Bayesian and Frequentist spatial methods to identify spatial variations of the disease risk and associated socioeconomic, demographic, and climatic factors for such spatial variations of the risk. While a wide variety of spatial and spatiotemporal methods have been employed since the beginning of this pandemic, we found that all of the spatial clustering studies had demonstrated spatial heterogeneity of COVID-19 risk. In almost all of the studies, retrospective data of all ages were used except for four studies that used specific vulnerable groups of the population. 

Among the frequentist methods, the global Moran’s *I* and local Moran’s *I* were the commonly used approach for identifying spatial clusters, followed by Getis-Ord GI* statistics and Kulldorff’s spatial scan statistic. The local spatial regression method GWR, a frequentist method, was frequently used to identify the association between the potential risk factors and COVID-19. A total of five studies used frequentist spatial statistical models such as spatial autoregressive models and GLM models to observe the spatial risk and associations. Four studies adopted frequentist spatial interpolation modelling approaches, whereas six studies have adopted a Bayesian spatial interpolation modelling approach to create smoothed surface risk map of COVID-19. 

Most of the studies used the frequentist approaches (85%), while only 15% of the studies used a Bayesian approach. Bayesian methods are often preferred over frequentist methods as the Bayesian approaches allow incorporating a wide range of components using a hierarchical modelling scheme that can allow a more robust assessment of the prediction uncertainties [198]. COVID-19 is often asymptomatic and under-reported globally, leading to instances of missing data at the national registries [199]. Bayesian methods have the advantage of accounting for these unreported or unobserved data or missing covariates by incorporating random effects into the model [19,200]. 

Various spatial and spatiotemporal models were used in 18 studies that used Bayesian hierarchical modelling to estimate the spatial risk and/or to identify the risk factors. The modelling framework of those models was dependent on the data type, distribution, outcome, and applications. Most Bayesian spatial or spatiotemporal models used a GLMM framework that includes fixed effects such as spatial, temporal, and spatiotemporal random effects. The Besag, York, and Mollié (BYM) model [184] was the most frequent global spatial smoothing specification used in this review. BYM provides easy implementation in a range of software. However, caution may be taken to minimize the potential over-smoothing of the BYM spatial models [201]. Future models can compare the impact of using other spatial smoothing priors [202]. First or second-order random walk terms were more commonly used for the temporal random effects. Gayawan et al., 2020 have used a P-spline model to allow nonlinear area-specific trends for the varying disease risk. The space-term interaction term, introduced by Knorr-held [189], was most commonly used for the space-time random effect. Most of the studies have used a Poisson-based modelling approach where data was assumed to have Poisson distribution. A novel separable Gaussian spatiotemporal model proposed by Rawat et al., 2021 [179] included an appropriately specified space-time process that provided an advantage of predicting the response variable for any spatial location and at any time point, even if it is unobserved within the data.

In our review, the INLA was the most adopted sampling method to fit the Bayesian models. INLA has recently become widely popular for its fast computational efficiency, which can provide accurate results in substantially less computing time [203]. INLA is an alternative to the traditional MCMC (the exact method for Bayesian inference) approaches which were only adopted in 4 studies in our review. However, INLA can fall short in recovering the true estimates for the random effects, their precisions, and model goodness of fit measures [204]. Future studies are warranted to compare the posterior estimates from both approaches. DIC and WAIC values were commonly used for model selection criteria to measure and compare the goodness-of-fit among different models and to select the best-fitted Bayesian models.

Sensitivity analysis of the priors is an integral part of the model validation process in Bayesian statistical modelling. However, only four studies in our review have tested the sensitivity of the priors. Future studies should incorporate a sensitivity analysis using alternate priors or hyperpriors in the final model to ensure the results are not sensitive to the prior specifications.

Another issue to be considered while modelling the spatial dynamics of COVID-19 is the spatial scale of analysis. Only 21% of the studies were conducted on a local spatial scale. COVID-19 risk may be sensitive to spatial differences at a local, regional, or global level. Since most of the studies used aggregated data, a local spatial scale analysis may produce a markedly different result than the regional or global spatial scale and increase the predictive accuracy and capacity. 

Our review shows that many studies found that the aging population and higher population density were the most influential factors in explaining the increased risk of COVID-19. Different ethnicity or minority status, income, education, and vulnerability index were also found to be associated with the risk of COVID-19. Among the climatic factors: temperature, relative humidity, land surface temperature and wind speed were commonly found to be associated with the spatial risk for COVID-19. These factors were recognized as important risk factors and should be incorporated into the risk modelling of COVID-19 in future. 

Our review highlights the flexibility and prominence of different geospatial methods in modelling the spatial risk of COVID-19 dynamics and understanding disease etiology. Using these spatial methods and tools can enable a more detailed view of the etiology of COVID-19 and allow faster and more reliable decision-making for the government or public health officials. The findings of this review can point to a few recommendations for researchers for improved practices and provide an opportunity for future application and development of spatial methods for COVID-19 studies. Given the potential benefits of Bayesian models to accommodate for the unreported or missing case data and missing variables, common issues in the COVID-19 risk mapping, we recommend more studies adopting this approach at the local spatial scale for improved predictive accuracy. Over 80% of the studies in our review used a short-term length of study period (1–6 months). More studies using a longer temporal dataset to observe the long-term impacts, patterns and trends of COVID-19 are also needed. 

A major strength of our review was that compared to the previous reviews [23,24], our review has qualitatively assessed the model structures, validation scheme, sampling methods, and sensitivity testing of the Bayesian models. The findings from this review show a positive trend in using spatial epidemiological tools by the scientific communities to understand the spatial transmission mechanism of COVID-19. Our review suggests that while the spatial and temporal analysis has been greatly applied, the quality of these studies and the analytical approaches varied by study. Our review provides a blueprint of existing work conducted in the field and reveals future research scope into advancing and developing spatial methods for studying COVID-19. Compared to the earlier review paper, this review also benefits from including a number of methodological limitations of existing spatial studies that can hinder the ability to provide sound evidence to guide local control efforts to reduce the burden of COVID-19. Finally, in the era of open data policy and reproducible research, this review emphasizes the importance of reviewing, validating and updating existing models to improve current research quality and the need for developing novel methodological approaches. Finally, we have adopted an exhaustive search strategy in accordance with the PRISMA guidelines, and therefore, we believe our review provided a fair representation of COVID-19 risk mapping efforts. 

Our review has a few limitations. It is acknowledged that despite the screening and extraction of data by two independent reviewers separately, it is possible that we may have excluded other papers relevant to our study objectives that may have provided valuable contributions. Since our review considered only those studies that were published in English, relevant articles published in other languages might also have been excluded. The included studies in our review adopted a diverse range of methodological approaches, therefore, performing a meta-analysis is out of the scope of this review. 

Finally, eradicating COVID-19 remains an ongoing challenge worldwide, and applying robust modelling tools will continue to be an important priority in global COVID-19 control and elimination efforts. There is a need to develop effective tools and advancing current technology in this field that can be useful for studying the spatial transmission of diseases that can help prevention of similar pandemics in future. It may be mentioned that there is an increasing number of detailed local data available to the researchers for COVID-19. For example, self-reported or crowdsourced data have the potential to provide real-time visualization of spatial clusters. The advancement of research efforts can include focusing on improving the precision and reliability of COVID-19 Bayesian model fitting using different types of neighborhood structures, proper and improper priors in spatial random effects, temporal random effects, and different types of space-time interactions. Studies on spatiotemporal analysis using point data could also provide strong evidence to support policy decision-making. Further advancement of reliable and robust modelling of COVID-19 will essentially depend on the acquisition and availability of good quality data with finer spatial and temporal resolutions and by taking account of the uncertainties in disease modelling. 

## Figures and Tables

**Table 1:** Spatial methods used for clustering and risk factor identification in COVID-19 studies.

Method Category	Method Name	No. of Articles N (%)	References
Frequentist Methods			
Spatial Clustering	Global Moran’s *I*	49 (31.8%)	[32,33,34,35,36,37,38,39,40,41,42,43,44,45,46,47,48,49,50,51,52,53,54,55,56,57,58,59,60,61,62,63,64,65,66,67,68,69,70,71,72,73,74,75,76,77,78,79,80]
	Local Moran’s *I* (LISA)	46 (29.8%)	[5,32,34,36,38,40,41,44,54,55,61,62,64,65,66,67,68,69,70,72,73,74,75,76,77,78,79,81,82,83,84,85,86,87,88,89,90,91,92,93,94,95,96,97,98,99]
	Average Nearest Neighbor (ANN)	2 (1.3%)	[54,80]
	Getis-Ord GI*statistic	36 (23.3%)	[5,29,33,35,41,46,48,49,54,55,56,58,59,60,61,67,69,74,76,77,80,84,88,100,101,102,103,104,105,106,107,108,109,110,111,112]
	Kernel Density Estimation	9 (5.8%)	[29,31,88,92,113,114,115,116,117]
	K-means Cluster	2 (1.3%)	[57,118]
	Ripley’s K function	1 (0.6%)	[72]
	Kulldorff’s spatial scan statistic	10 (6.5%)	[39,42,67,119,120,121,122,123,124,125]
Spatiotemporal Clustering	Kulldorff’s space-time scan statistic	24 (15.5%)	[4,5,31,40,44,52,59,92,100,126,127,128,129,130,131,132,133,134,135,136,137,138,139,140]
	MST-DBSCAN	1 (0.6%)	[124]
	Spatiotemporal event sequence-based clustering	1 (0.6%)	[139]
Spatial Regression	Spatial Regression Models (SEM/SLM)	20 (13%)	[45,51,53,65,76,87,91,94,95,119,132,141,142,143,144,145,146,147,148,149]
	Geographically Weighted Regression	36 (23.3%)	[5,38,47,49,56,58,62,71,72,76,79,80,86,87,90,98,99,105,108,116,118,140,141,143,144,146,150,151,152,153,154,155,156,157,158,159]
	Geodetector Q statistic	4 (2.6%)	[66,68,160,161]
Spatial Statistical Modeling	Spatial autoregressive (SAR)	1 (0.6%)	[64]
	GLM Regression model	1 (0.6%)	[118]
Spatiotemporal Statistical Modeling	Poisson-based Distributed lagged nonlinear model with a spatial function	1 (0.6%)	[162]
	Generalized additive model	2 (1.3%)	[43,163]
Spatial Interpolation	Areal Interpolation	1 (0.6%)	[151]
	Inverse distance weighting (IDW)	2 (1.3%)	[107,164]
	Thiessen Polygon method	1 (0.6%)	[165]
Bayesian Methods			
Spatial Interpolation	Local empirical Bayesian Smoothing	6 (3.9%)	[38,62,78,83,94,113]
Spatial Statistical Modeling	GLMM Spatial models	5 (3.2%)	[166,167,168,169,170]
Spatiotemporal Statistical Modeling	GLMM spatiotemporal models	11 (7.1%)	[112,160,171,172,173,174,175,176,177,178,179]
	Geo-additive hurdle Poisson spatiotemporal model	1 (0.6%)	[180]
	Bayesian Model Averaging	1 (0.6%)	[181]

**Table 2:** Structure of the Bayesian statistical models.

Reference	Model	Space	Time	Space-Time	Model Validation	Bayesian Inference
Bermudi et al., 2021 [171]	Poisson latent Gaussian Bayesian model	BYM	RW (1)	Space-time interaction term	DIC	INLA
Blangiardo et al., 2020 [172]	Poisson Bayesian hierarchical model	BYM	RW (1), RW (2)	__	__	INLA
Briz-Redón et al., 2022 [173]	Poisson based Bayesian hierarchical model	BYM	RW (2)	Space-time interaction term	DIC and WAIC	INLA
Lima et al., 2021 [166]	Poisson Bayesian SAM	BYM	__	__	DIC and WAIC	INLA
DiMaggio et al., 2020 [167]	Poisson Bayesian hierarchical model	BYM	__	__	DIC	INLA
Gayawan et al., 2020 [180]	Geo-additive hurdle Poisson model	BYM	P-spline	Space-time interaction term	DIC	MCMC
Jalilian et al., 2021 [174]	Poisson Bayesian hierarchical model	BYM	RW (2)	__	DIC, WAIC and BCV	INLA
Jaya et al., 2021 [175]	Poisson Bayesian hierarchical model	Leroux CAR	RW (1), RW (2)	Space-time interaction term	DIC and WAIC	INLA
Johnson et al., 2021 [176]	Poisson Bayesian hierarchical model	BYM	RW (1)	Space-time interaction term	DIC	INLA
Ngwira et al., 2021 [177]	Poisson Space-time inseperable model	BYM	RW (1), RW (2)	Space-time interaction term	DIC	INLA
Olmo et al., 2021 [181]	Bayesian Model Averaging	Autoregressive lagged spatial terms	Autoregressive lagged terms	__	HPM and BPM	MCMC
Paul et al., 2021 [178]	Bayesian semi-parametric spatiotemporal Negative Binomial model	ICAR	RW (1)	With zero-mean Gaussian distribution	WAIC	INLA
Paul et al., 2020 [112]	Bayesian Spatiotemporal Model	__	__	Latent Gaussian	__	MCMC
Rawat et al., 2021 [179]	Bayesian separable Gaussian spatiotemporal model	Exponentially decaying pattern	Exponentially decaying pattern	Gaussian process with zero mean	MAPE, RMSE, CRPS	INLA
Wang et al., 2021 [160]	Poisson Bayesian hierarchical model	Spatial term	Gaussian noise	Space-time interaction effect	__	MCMC
Whittle et al., 2020 [168]	Poisson Bayesian hierarchical model	BYM2	__	__	DIC	INLA
Millett et al., 2020 [169]	Zero-inflated negative binomial model	BYM	__	__	__	INLA
Yang et al., 2021 [170]	Bayesian negative binomial hierarchical model	BYM	__	__	DIC	INLA

BYM: Besag–York–Mollié model; INLA: Integrated Nested Laplace Approximation; MCMC: Markov Chain Monte Carlo; DIC: Deviance Information Criterion; RW: Random Walk; WAIC: Watanabe–Akaike Information Criterion; SAM: Spatial autoregressive model; IWLS: Iterative Weighted Least Square; BCV: Bayesian cross-validation criterion; HPM: Highest probability model; BPM: Bayesian Purity Model; MAPE: mean absolute percentage error; RMSE: Root Mean Squared Error; CRPS: Continuous Ranked Probability Score.

**Table 3:** Risk factors for spatial variations of COVID-19.

Indicator	Risk Factors	No. of Studies (+,− Association)	References	Risk Factors	No. of Studies (+,− Association)	References
Demographic	%Asian	3 (2,1)	[45,64,142]	Aging population	21 (15,6)	[42,43,45,47,90,94,105,108,116,118,143,147,148,151,155,156,157,170,176,177,181]
	%Black	12 (12,0)	[45,51,64,108,112,119,142,149,151,168,169,178]	Middle Age population	2 (2,0)	[112,140]
	%Black female	1 (1,0)	[144]	Young population	1 (1,0)	[168]
	%Disabled population	1 (1,0)	[119]	BIPOC	1 (1, 0)	[49]
	%Hispanic	3 (3,0)	[51,142,149]	Ethnic minority	2 (2,0)	[147,170]
	%Native American	3 (3,0)	[142,149,158]	Immigrants	2 (2,0)	[42,118]
	%Urban population	1 (1,0)	[145]	English proficiency	2 (2,0)	[119,157]
	% White	1 (0,1)	[168]	Migration	2 (1,1)	[141,152]
	%Non-White	1 (1,0)	[176]	Population density	22 (22,0)	[5,38,42,47,53,65,80,86,91,95,98,105,118,142,143,146,148,149,153,156,160,168]
	Population size	2 (2,0)	[37,118,155,181]	Immigrants	1 (1,0)	[151]
	Ethnic minority	3 (3,0)	[141,150,154]	Lower Education	1 (1,0)	[176]
Socioeconomic	Deprivation Index	2 (2,0)	[53,151]	Income	9 (5,4)	[38,71,76,140,141,144,154,168,181]
	GDP	3 (1,2)	[148,159,160]	Poor housing	4 (2,2)	[51,150,158,176]
	GINI Index	2 (2,0)	[62,132]	Poverty level	4 (1,3)	[47,147,153,177]
	Health expenditures	1 (1,0)	[47]	Social Vulnerability Index	2 (2,0)	[65,87]
	Higher education	3 (0,3)	[151,155,178]	Spatial interaction index	1 (1,0)	[118]
	Unemployment rate	4 (4,0)	[64,71,149,178]	Total purchase power index	1 (1,0)	[118]
Climatic	Precipitation	3 (2,1)	[58,162,176]	Water vapor	1 (0,1)	[153]
	Relative humidity	3 (2,1)	[58,64,162]	Wind pressure	1 (1,0)	[153]
	Rainfall	1 (1,0)	[153]	Wind speed	3 (2,1)	[56,58,153]
	Temperature	5 (3,2)	[56,94,161,162,176]	LST	2 (1,1)	[153,176]

## Data Availability

All data generated or analysed during this study are included in this published article and its Supplementary Information Files.

## Supplementary Materials

The following supporting information can be downloaded at: https://www.mdpi.com/article/10.3390/ijerph19148267/s1, File S1: Study Details and Methods; File S2: Regression Data; File S3: Risk of Bias_JBI.

## Abbreviations

BAS	Bayesian Adaptive Sampling
BCV	Bayesian cross-validation criterion
BPM	Bayesian Purity Model
BYM	Besag–York–Mollié model
CAR	Conditional autoregressive
CRPS	Continuous Ranked Probability Score
DIC	Deviance Information Criterion
GIS	Geographic information system
GLMM	Generalized linear mixed models
GWR	Geographically weighted regression
HPM	Highest probability model
INLA	Integrated nested Laplace approximations
IWLS	Iterative Weighted Least Square
JBI	Joanna Briggs Institute (JBI)
PRISMA	Preferred reporting items for systematic review and meta-analysis
MAPE	Mean Absolute Percentage Error
MCMC	Markov chain Monte Carlo
RMSE	Root Mean Squared Error
RW	Random Walk
SAM	Spatial autoregressive model
WAIC	Watanabe–Akaike Information Criterion

## Appendix A

### Appendix A.1. Search Strings

Search terms used in Embase, Medline, Scopus, and Web of Science.

### Appendix A.2. Embase

1. sars-related coronavirus
2. (coronavirinae/ or betacoronavirus/ or coronavirus infection/) and (epidemic/ or pandemic/)
3. (nCoV* or 2019nCoV or 19nCoV or COVID19* or COVID or SARS-COV-2 or SARSCOV-2 or SARS-COV2 or SARSCOV2 or Severe Acute Respiratory Syndrome Coronavirus 2 or Severe Acute Respiratory Syndrome Corona Virus 2)
4. ((new or novel or “19” or “2019” or Wuhan or Hubei or China or Chinese) adj3 (coronavirus* or corona virus* or betacoronavirus* or CoV or HCoV))
5. ((coronavirus* or corona virus* or betacoronavirus*) adj3 (pandemic* or epidemic* or outbreak* or crisis))
6. ((Wuhan or Hubei) adj5 pneumonia)
7. or/1-6
8. limit 7 to yr = ”2019 -Current”
9. (Space-time clustering

OR spati*regres*.mp
OR spat* temp* pattern*.mp OR
geography* distribut*.mp OR spat* temp*
distribut*.mp OR heterogen* distribut.mp OR
spacetime cluster*mp OR space-time cluster*mp
OR hotspot.mp Or hot spots. mp OR geographically weighted regression OR cluster analysis OR spatial autocorrelation analysis OR GWR OR GIS OR geographic Information Systems)

10. 8 AND 9

### Appendix A.3. Medline

1. (coronavirus/ or betacoronavirus/ or coronavirus infections/) and (disease outbreaks/ or epidemics/ or pandemics/)
2. (nCoV* or 2019nCoV or 19nCoV or COVID19* or COVID or SARS-COV-2 or SARSCOV-2 or SARSCOV2 or Severe Acute Respiratory Syndrome Coronavirus 2 or Severe Acute Respiratory Syndrome Corona Virus 2)
3. ((new or novel or “19” or “2019” or Wuhan or Hubei or China or Chinese) adj3 (coronavirus* or corona virus* or betacoronavirus* or CoV or HCoV))
4. ((coronavirus* or corona virus* or betacoronavirus*) adj3 (pandemic* or epidemic* or outbreak* or crisis))
5. ((Wuhan or Hubei) adj5 pneumonia)
6. or/1-5
7. limit 6 to yr = ”2019 -Current”
8. (Space-time clustering

OR spati*regres*.mp
OR spat* temp* pattern*.mp OR
geography* distribut*.mp OR spat* temp*
distribut*.mp OR heterogen* distribut.mp OR
spacetime cluster*mp OR space-time cluster*mp
OR hotspot.mp Or hot spots. mp OR geographically weighted regression OR cluster analysis OR spatial autocorrelation analysis OR GWR OR GIS OR geographic Information Systems)

9. 7 AND 8

### Appendix A.4. Scopus

**Keywords** (coronavirus OR betacoronavirus OR “coronavirus infections”) AND (“disease outbreaks” OR epidemics OR pandemics) OR

**Title abs key** ncov* OR 2019ncov OR 19ncov OR covid19* OR covid OR sars-cov-2 OR sars-cov2 OR sarscov-2 OR sarscov2 OR “Severe Acute Respiratory Syndrome Coronavirus 2” OR “Severe Acute Respiratory Syndrome Corona Virus 2” OR (new W/3 coronavirus*) OR (new W/3 “corona virus*”) OR (new W/3 betacoronavirus*) OR (new W/3 cov) OR (new W/3 hcov) OR (novel W/3 coronavirus*) OR (novel W/3 “corona virus*”) OR (novel W/3 betacoronavirus*) OR (novel W/3 cov) OR (novel W/3 hcov) OR (19 W/3 coronavirus*) OR (19 W/3 “corona virus*”) OR (19 W/3 betacoronavirus*) OR (19 W/3 cov) OR (19 W/3 hcov) OR (2019 W/3 coronavirus*) OR (2019 W/3 “corona virus*”) OR (2019 W/3 betacoronavirus*) OR (2019 W/3 cov) OR (2019 W/3 hcov) OR (wuhan W/3 coronavirus*) OR (wuhan W/3 “corona virus*”) OR (wuhan W/3 betacoronavirus*) OR (wuhan W/3 cov) OR (wuhan W/3 hcov) OR (hubei W/3 coronavirus*) OR (hubei W/3 “corona virus*”) OR (hubei W/3 betacoronavirus*) OR (hubei W/3 cov) OR (hubei W/3 hcov) OR (china W/3 coronavirus*) OR (china W/3 “corona virus*”) OR (china W/3 betacoronavirus*) OR (china W/3 cov) OR (china W/3 hcov) OR (chinese W/3 coronavirus*) OR (chinese W/3 “corona virus*”) OR (chinese W/3 betacoronavirus*) OR (chinese W/3 cov) OR (chinese W/3 hcov) OR (coronavirus* W/3 outbreak*) OR (coronavirus* W/3 crisis) OR (“corona virus*” W/3 pandemic*) OR (“corona virus*” W/3 epidemic*) OR (“corona virus*” W/3 outbreak*) OR (“corona virus*” W/3 crisis) OR (betacoronavirus* W/3 pandemic*) OR (betacoronavirus* W/3 epidemic*) OR (betacoronavirus* W/3 outbreak*) OR (betacoronavirus* W/3 crisis) OR (wuhan W/5 pneumonia) OR (hubei W/5 pneumonia) **AND** (spat* temp* pattern*) OR (geography* distribut*) OR (spat* temp* distribut*) OR (heterogen* distribut*) OR (spacetime cluster*) OR (space-time cluster*) OR hotspot Or hot spots OR (geographically weighted regression) OR (cluster analysis) OR (spatial autocorrelation analysis) OR GWR OR GIS OR (geographic Information Systems) or (Spatial analysis) OR (Spatiotemporal analysis) OR (Geographic Information System) OR (geographic Mapping) OR (geographic distribution) OR (spatial regression) OR (spatial autocorrelation) OR (Spatiotemporal analysis) OR (clustering analysis) OR (spatiotemporal analysis).

### Appendix A.5. Web of Science

“Wuhan coronavirus” OR “COVID19*” OR “COVID-19*” OR “COVID-2019*” OR “coronavirus disease 2019” OR “SARS-CoV-2” OR “2019-nCoV” OR “2019 novel coronavirus” OR “severe acute respiratory syndrome coronavirus 2” OR “2019 novel coronavirus infection” OR “coronavirus disease 2019” OR “coronavirus disease-19” OR “SARS-CoV-2019” OR “SARS-CoV-19”.

### Appendix A.6. AND

(Spatial cluster) OR
(Spatial hotspot)
(Spatiotemporal hotspot) OR
(Spatiotemporal cluster)
(Geographic Mapping) OR
(geographic distribution) OR
(spatial regression) OR
(spatial autocorrelation analysis) OR
(Spatiotemporal analysis) OR
(hotspot) OR (geographically weighted regression) OR (Clustering analysis)
